# Modeling the potential global distribution of suitable habitat for the biological control agent *Heterorhabditis indica*

**DOI:** 10.1002/ece3.8997

**Published:** 2022-06-10

**Authors:** Sumeet Kour, Uma Khurma, Gilianne Brodie, Sunil Singh

**Affiliations:** ^1^ School of Agricultural, Geography, Environment Ocean and Natural Science The University of the South Pacific Suva Fiji Islands; ^2^ Institute of Applied Sciences The University of the South Pacific Suva Fiji Islands; ^3^ School of Pacific Arts, Communication and Education The University of the South Pacific Suva Fiji Islands

**Keywords:** biological control, CLIMEX, ecological niche model, entomopathogenic nematode, *Heterorhabditis indica*, potential global distribution

## Abstract

Entomopathogenic nematode (EPN) *Heterorhabditis indica* is a promising biocontrol candidate. Despite the acknowledged importance of EPN in pest control, no extensive data sets or maps have been developed on their distribution at global level. This study is the first attempt to generate Ecological Niche Models (ENM) for *H*. *indica* and its global Habitat Suitability Map (HSM) for *H. indica* to generate biogeographical information and predicts its global geographical range and help identify of prospective areas for its exploration and to help identify the suitable release areas for biocontrol purpose. The aim of the modeling exercise was to access the influence of temperature and soil moisture on the biogeographical patterns of *H*. *indica* at the global level. Global *Heterorhabditis indica* ecosystems. CLIMEX software was used to model the distribution of *H*. *indica* and assess the influence of environmental variable on its global distribution. In total, 162 records of *H*. *indica* occurrence from 27 countries over 25 years were combined to generate the known distribution data. The model was further fine‐tuned using the direct experimental observations of the *H*. *indica's* growth response to temperature and soil moisture. Model predicts that much of the tropics and subtropics have suitable climatic conditions for *H*. *indica*. It further predicts that *H*. *indica* distribution can extend into warmer temperate climates. Examination of the model output, predictions maps at a global level indicate that *H*. *indica* distribution may be limited by cold stress, heat stress, and dry stresses in different areas. However, cold stress appears to be the major limiting factor. This study highlighted an efficient way to construct HSM for EPN potentially useful in the search/release of target species in new locations. The study showed that *H*. *indica* which is known as warm adapted EPN generally found in tropics and subtropics can potentially establish itself in warmer temperate climates as well. The model can also be used to decide the release timing of EPN by adjusting with season for maximum growth. The model developed in this study clearly identified the value and potential of Habitat Suitability Map (HSM) in planning of future surveys and application of *H*. *indica*.

## Graphical abstract

This study, highlighted an efficient way to construct habitat suitability map (HSM) for entomopathogenic nematode (EPN) potentially useful in the search/release of target species in new locations. The study showed that *Heterorhabditis indica* which is known as warm adapted EPN generally found in tropics and subtropics can potentially establish itself in warmer temperate climates as well. The model can also be used to decide the release timing of EPN by adjusting with season for maximum growth. The model developed in this study clearly identified the value and potential of HSM in planning of future surveys and application of *H. indica*.

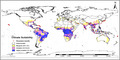

## 1 INTRODUCTION

Entomopathogenic nematodes (EPN) have proved useful in controlling insect pests belonging to order Coleoptera, Lepidoptera, and Diptera and have the potential to replace costly and hazardous chemical pesticides to an extent or be an effective option in Integrated Pest Management (IPM) programs (Georgis et al., 2006). Extensive surveys have been conducted across the globe to isolate EPN species and exploit them as biocontrol agent of soil‐dwelling and foliar insect pests in agricultural fields. Till date, around 121 valid species of entomopathogenic nematode (EPN) belonging to genera *Steinernema* (100 species) and *Heterorhabditis* (21 species) have been identified from different countries of the world (Bhat et al., 2020). For economic and successful use, it is important that the naturally occurring species of biocontrol agents are discovered and tested against high priority local crop pests. Also, it is widely accepted that locally collected EPN should be released for biocontrol as this would exclude many risks associated with introducing exotic species into new environments (Abate et al., 2017; Askary et al., 2018; Noosidum et al., 2010). Hence, search for EPN in their natural habitat is the first step toward their use in pest control programs (Slininger et al., 2003). Even though EPN are ubiquitous in nature (Hominick, 2002), the recovery frequency during surveys conducted worldwide is usually low. EPN recovery from the field requires considerable resources, expertise, and time. The search for suitable EPN to employ as biocontrol agent may become more efficient if geographical distribution of target EPN species is known. Despite the acknowledged importance of EPN in pest control (Askary & Abd‐Elgawad, 2017, 2021; Lacey & Georgis, 2012), no extensive data sets or maps have been developed on their distribution at country, regional, or global level. For many of the described EPN species, only a few exact localities with geographic coordinates have been published and their occurrence is generally presented in the form of point maps. Such point maps show the occurrence data but convey no information on the likelihood of their occurrence in nonsurveyed areas. It is, therefore, difficult to prioritize search areas for successful isolation of EPN and to effectively plan their use in biocontrol programs. In such cases, Habitat Suitability Maps (HSM) presenting potential habitats where the species are likely to be found, can help in prioritizing areas for searching and increase chances of successful isolation of EPNs. HSM can also help in identifying potential habitats where species can establish itself post release. Ecological Niche Models (ENM) are frequently used to predict geographic distribution of suitable habitats of modeled species and to generate their HSM (Franklin, 2010; Mukherjee et al., 2011). ENM is a GIS‐based method that commonly utilizes the information on climatic tolerances and ecology of an organism in their native habitat, to identify habitats in other geographical regions where populations could potentially occur (Pearson, 2010). This approach has proven valuable for generating biogeographical information and predicts the geographical range of a species without conducting extensive surveys (Steinbauer et al., 2002). CLIMEX software has proved useful in biocontrol research, in identification of prospective areas for the exploration and to study post release establishment patterns of biological control agents (Firehun et al., 2013; Goolsby et al., 2005; Japoshvili et al., 2015; Lawson et al., 2010; Senaratne et al., 2006; Shrestha et al., 2015; Zalucki & Van Klinken, 2006). This software has found to be ideal for modeling the species with patchy distribution and point distribution record (Hill & Thomson, 2015) and can be used to model EPN species distribution. CLIMEX models have also been widely used for many pests including a few nematode species and have been useful in projecting their distributions (Boag et al., 1997; Singh et al., 2021; Yeates et al., 1998). CLIMEX model makes use of both species’ distribution and phenology data (temperature, moisture preferences, wet, cold, heat, and dry stresses) and correlates with metrological datasets to make predictions. Thus, the HSM generated provides a scientific basis for identifying areas where there is high likelihood of species being found or has the potential to successfully establish itself. However, CLIMEX or any other simulation software has never been used for modeling the distribution of any EPN species.

Despite 155 published records of *H*. *indica's* occurrence from 27 countries, there is no species distribution map at global level. Therefore, this study develops a predictive model of the likely global distribution of *H*. *indica* and its suitable habitat. It is believed that this approach can be both economic and time efficient. The aim of the modeling exercise of this study was to develop an ecological niche model and to generate HSM for *H*. *indica* using the CLIMEX software to know *H*. *indica's* possible global geographic range to better design the future surveys and to help identify the suitable release areas. The study also aimed to investigate the importance of environmental variables when projecting the range and spatial pattern of the modeled EPN species. These methods may also be used for modeling the distribution of other EPN species and to make better informed decisions while using EPN as biocontrol agents.

## 2 MATERIALS AND METHODS

CLIMEX software version 4.0 (Hearne Scientific Software Company) for windows was used to model the distribution of potential biocontrol agent *H*. *indica* under current climatic conditions. “Compare Locations model” for single species was utilized for the purposes of this study. Annual growth index (GI) was calculated as a function of soil moisture and temperature, and stress indices (cold, wet, hot and dry) were used to determine the probability that the population can survive in unfavorable conditions. To give an overall measure of favorability of a locality for permanent occupation by the population, growth indices, and stress indices are calculated weekly and combined to give “Ecoclimatic Index” (EI), an annual index of climatic suitability. In this study, geographic locations with EI > 20 represent very suitable climatic conditions for establishment, survival, and growth of *H*. *indica*. Locations with EI values between 10 and 19 are considered suitable and conducive to *H*. *indica* survival; and locations with EI values less than 10 are considered marginally favorable for establishment and survival of the species (Ganley et al., 2009). The model was parameterized using global distribution of *H*. *indica* and published laboratory derived ecological study results. The parameters were iteratively adjusted depending on satisfactory agreement between the potential and known worldwide distribution of *H*. *indica*. The parameters were subsequently verified by comparing to ecological and physiological data of *H*. *indica* to ensure that they were biologically reasonable. ArcMap version 10.4.1 (Minami, 2000) was used to map CLIMEX output.

### 2.1 *Heterorhabditis indica* occurrence data

In total, 155 records of *H*. *indica* occurrence from published research papers and 7 records from online database Global Biodiversity Information Facility database (GBIF) (http://www.gbif.org) were combined to generate the known distribution data. Not all published research papers provided exact geographic coordinates for positive sites so in those cases the Google Earth (https://www.google.com/earth/) and the Google Maps (https://www.google.com/maps/preview) were used to best determine coordinates for the occurrence site. Duplicate records were removed.

### 2.2 Climate data

The CliMond CM10_1975H_V1 interpolated climate surface available at https://www.climond.org/CLIMEX.aspx was used while running CLIMEX following Kriticos et al. (2012). CliMond CM10_1975H_V1 is fine scale 10 arc minute (~18 km) dataset which has long‐term (1950–2000) monthly climate means, centered on 1975 for maximum temperature (T max), minimum temperature (T min), precipitation (P total), and relative humidity at 9 a.m. (RH09:00) and 3 p.m. (RH15:00). Climate classification by Koppen‐Geiger (KG) was used in this study (Kottek et al., 2006).

### 2.3 Model fitting and verifications

Since *H*. *indica* occurs primarily in tropical and sub‐tropical climatic conditions, the Wet Tropical template provided with CLIMEX software was selected to create the “species parameter file.” The model was calibrated using species‐specific physiological tolerance thresholds. The climatic requirements of *H*. *indica* are inferred from its current known occurrence. Developmental threshold temperature data of *H*. *indica* and moisture requirement was inferred from laboratory data given by Kour et al. (2021) and were used to define parameter values for temperature index and moisture index. Values for stress indices were adjusted iteratively, by running and re‐running the parameter file with different parameter values so that the projected climate suitability patterns match with the known occurrence of *H*. *indica*. The parameter values used in this study to model *H*. *indica* are given in Table 1. After every iteration, the distribution map predicted by the model output was compared with the observed distribution map of *H*. *indica*. The best fit was then used to project *H*. *indica* potential global distribution.

**TABLE 1:** Parameters used in the CLIMEX model for *Heterorhabditis indica*

Index	Parameter	Value
Temperature (℃)	DV0 = limiting low temperature	18
	DV1 = lower optimum temperature	25
	DV2 = upper optimum temperature	30
	DV3 = limiting high temperature	33
Moisture	SM0 = limiting low soil moisture	0.08
	SM1 = lower optimum soil moisture	0.1
	SM2 = upper optimum soil moisture	0.7
	SM3 = limiting high soil moisture	1.7
Cold stress	TTCS = temperature threshold	11
	THCS = stress accumulation rate	0.0002
Heat stress	TTHS = temperature threshold	36
	THHS = stress accumulation rate	0.0003
Wet stress	SMWS = wet stress threshold	1.7
	HWS = wet stress accumulation rate	0.003
Dry stress	SMDS = dry stress threshold	0.08
	HDS = dry stress accumulation rate	0.001

### 2.4 Temperature index

The lower (DV0) and upper (DV3) temperature thresholds for growth, after which species stop growing, were set to 18 and 33°C, respectively (Table 1). Initially, DV0 was set at 20°C. Setting DV0 at 20°C did not predict *H*. *indica* occurrence in Alexandria, Egypt; hence it was reduced to 18°C to include the positive site in Alexandria, Egypt. The lower optimum temperature threshold (DV1) and upper optimum temperature threshold (DV2) were set at 25 and 30°C, respectively, as per the optimum temperature regime established in ecological studies reported by Kour et al. (2021). Temperature above 35°C is lethal to *H*. *indica* (Kour et al., 2021); hence DV3 was set to 33°C. The temperature range of 18–33°C could represent all the recorded distribution of *H*. *indica* except one. The temperature range is also in agreement with the thermal niche breadth of *H*. *indica* found in the ecological studies reported by Kour et al. (2021).

### 2.5 Moisture index

A value of SM = 0 indicates no soil moisture; SM = 0.5 indicates soil moisture content is 50% of Held capacity; SM = 1 indicates that the soil moisture content is 100% of capacity (Sutherst et al., 2007). To allow reasonable species growth, SM0 was set at 0.08. Soil moisture values for optimum growth (SM1 and SM2) were set at 0.1 and 0.7, respectively, to fit observed occurrence of species in United Arab Emeritus, Palestine, and Egypt. An upper soil moisture level (SM3) of 1.7 was adapted following a similar approach.

### 2.6 Cold stress

The cold stress temperature threshold (TTCS) mechanism was used to describe the response of *H*. *indica* to cold. The TTCS procedure assumes that stress will start below a given temperature and accumulate at a given weekly rate. The cold stress was calculated as per the method described by Pivonia and Yang (2004). The following equation was used to calculate weekly cold stress: weekly stress = (THCS) × (TTCS) average weekly min temperature) × number of weeks, where THCS is the rate at which cold stress accumulates when minimum temperature is less than TTCS and number of weeks stands for the successive number of weeks with stress. THCS was calculated using the maximum time permitted between two infections to maintain a population. In soil, *H*. *indica* can survive for 7–8 months before they find a new host (Mauleon et al., 2006). Hence, the maximum time permitted between two infection events was estimated to be 36 weeks. TTCS was set at 11°C and THCS at 0.0002. This rate provided an appropriate fit to the observed occurrence of *H*. *indica* and ensured that the sites in the cold coastal region of Alexandria, northern Egypt and in warm temperate coastal region of Japan remained in the model, but cooler regions are excluded.

### 2.7 Heat stress

Temperature is one of the most important factors affecting the infectivity and survival of infective juveniles (IJ). Temperatures above 35°C are sub lethal and above 40°C is lethal for IJ (Sandouka et al., 2003). To estimate heat stress, the method described by Pivonia and Yang (2004) was followed. Heat stress temperature threshold (TTHS) was set at 36°C and the weekly rate (THHS) was set at 0.0003 to allow year‐round survival to *H*. *indica* population in hot climate along coastal region of United Arab Emirates. These values provided an appropriate fit to the observed global occurrence of *H*. *indica*.

### 2.8 Dry stress

EPN are very much dependent on moisture availability for survival and movement. The dry stress parameter (SMDS) was set at the same level (0.08) as the lower soil moisture threshold (SM0) because soil moisture‐related stress probably begins at the same soil moisture levels where growth stops. The stress accumulation rate of 0.001 per week was set to include the occurrence of the *H*. *indica* in drier areas in United Arab Emirates from where it has been reported.

### 2.9 Wet stress

The wet stress parameter was fitted through iteration where parameter was adjusted to match the known occurrence of *H*. *indica*. The threshold value for wet stress (SMWS) of 1.7 with an accumulation rate (HWS) of 0.003 was used.

### 2.10 Model verification

In the final development phase, CLIMEX model was verified qualitatively by evaluating its ability to predict currently known occurrence of *H indica*. Occurrence with EI values > 0 were interpreted as correctly predicted presence. Following verification, the parameter file was run using the "Compare Locations" function to examine predicted global distributions.

## 3 RESULTS

### 3.1 Model fitting and verification

**FIGURE 1:**
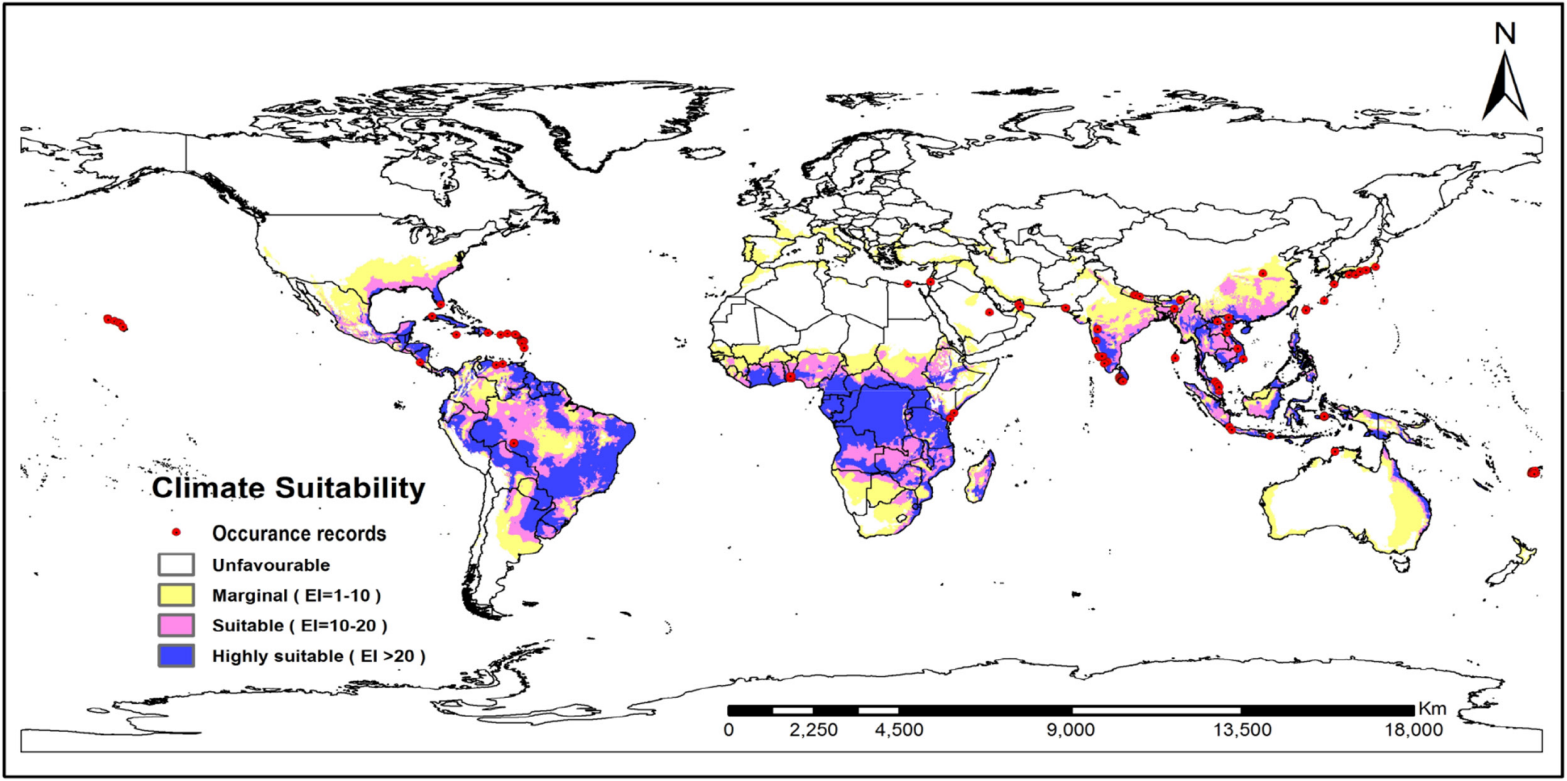
Ecoclimatic suitability (EI) for *Heterorhabditis indica*, under the current climate scenario as predicted using CLIMEX. Red circles indicate known recorded occurrence of *H*. *indica*

The current recorded global occurrence of *H*. *indica* includes 27 countries and island groups between 20°S and 40°N (Figure 1). Under a current climatic scenario, the predicted global distribution of *H*. *indica* shows a very close agreement with the known geographic range of *H*. *indica* (Figure 1). Occurrence records accord well with the modeled climate suitability for the area, and the present distribution is consistent with the EI values except for one occurrence record in Saudi Arabia which falls in the region predicted to be unsuitable for its occurrence.

### 3.2 Model projection at global level

**FIGURE 2:**
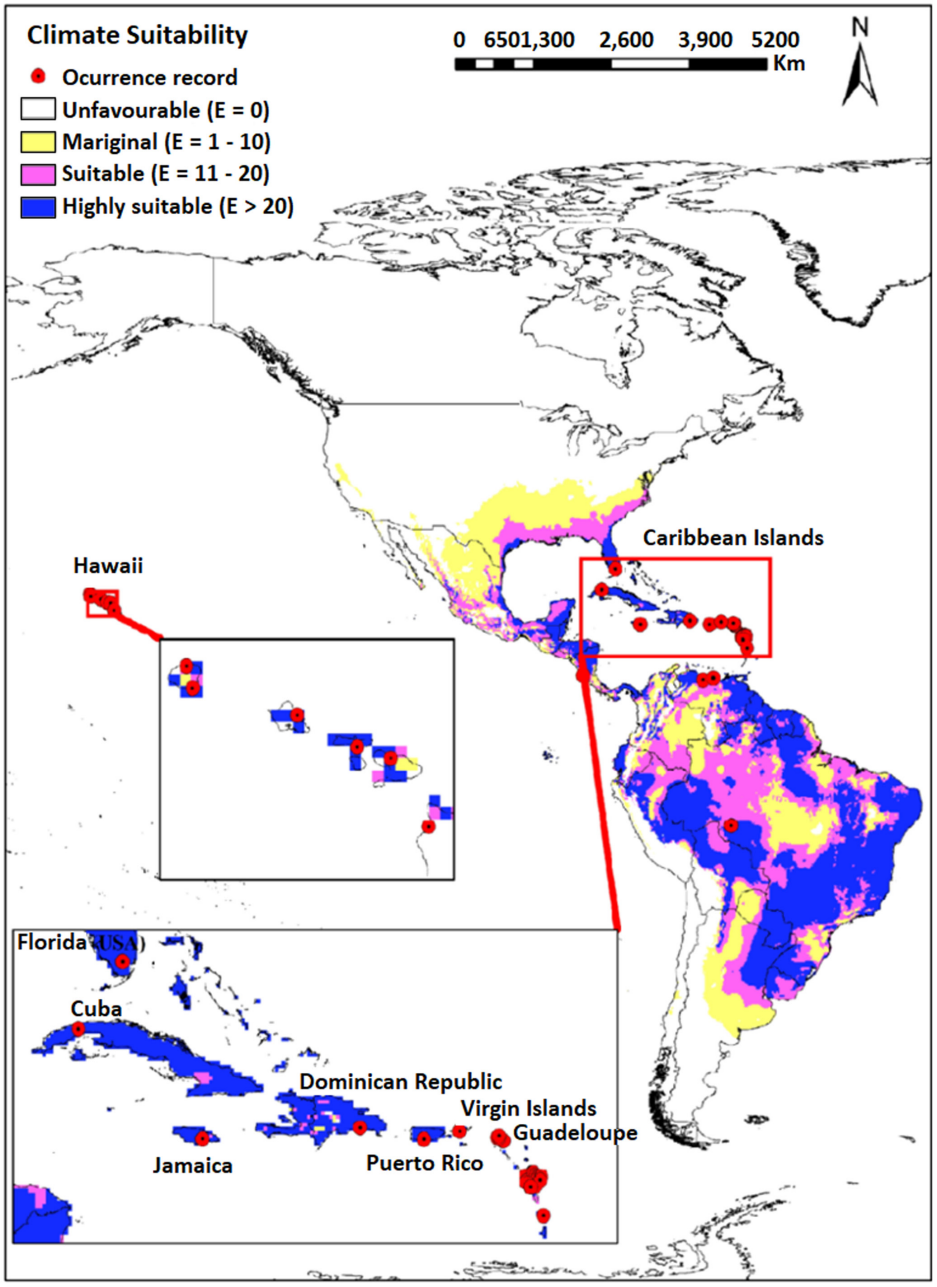
Ecoclimatic suitability (EI) for the *Heterorhabditis indica* in North and South America under the current climate scenario as predicted using CLIMEX. Red circles indicate recorded occurrence of *H*. *indica*

**FIGURE 3:**
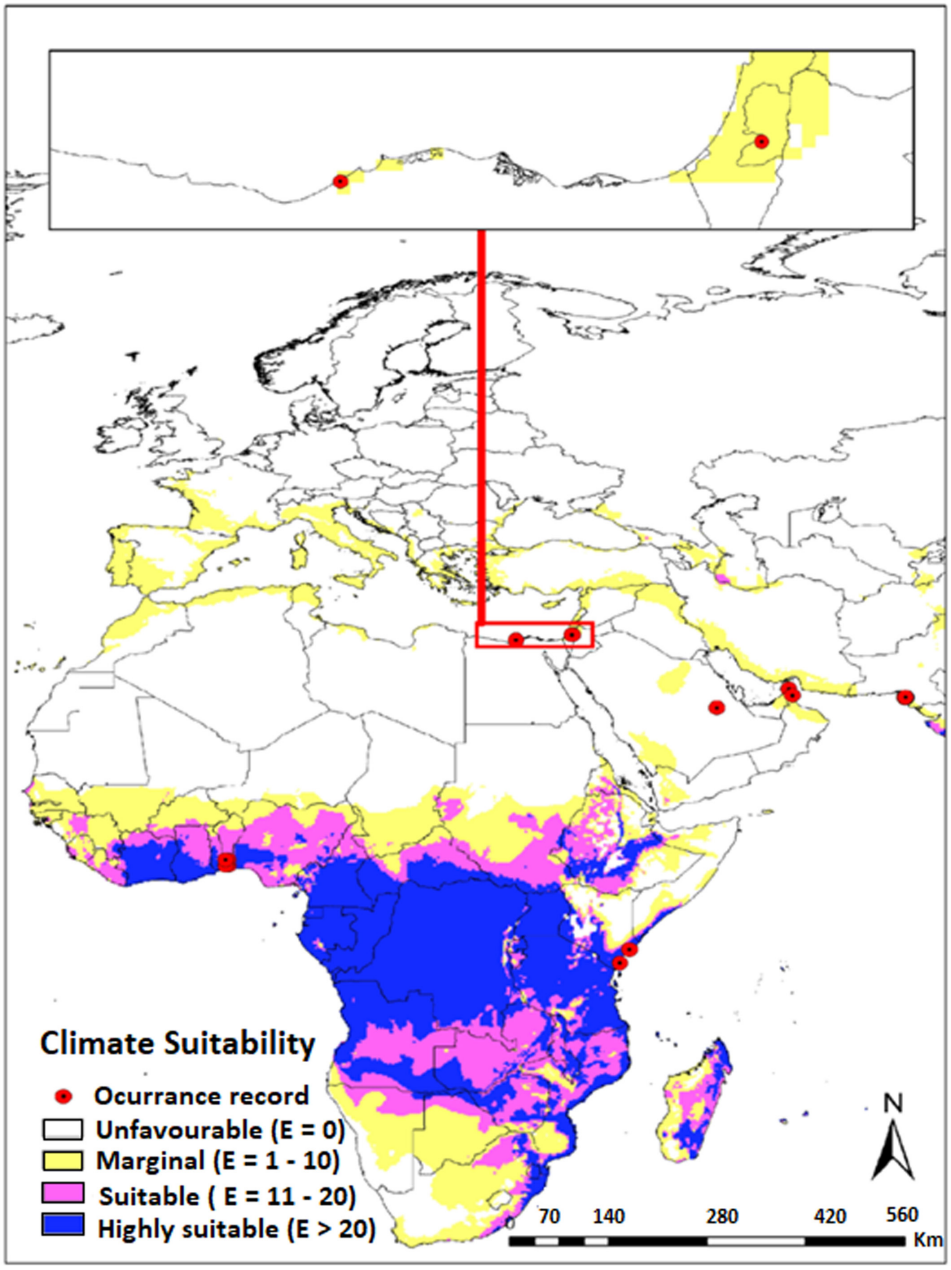
Ecoclimatic suitability (EI) for the *Heterorhabditis indica* in Europe and Africa under the current climate scenario as predicted using CLIMEX. Red circles indicate recorded occurrence of *H*. *indica*

In the map obtained in this study, much of the tropics and subtropics are predicted to have moderately suitable or highly suitable climatic conditions for *H*. *indica*. The predicted distribution also extends into warmer temperate climates, such as those found in the coastal regions of southern Europe. All Caribbean islands (EI = 41–83) and a large portion of Central America that is, Honduras, Nicaragua, Belize, eastern Panama, lowland Petén, and the eastern coast of Guatemala are predicted to also have highly suitable climate (EI = 29–51) (Figure 2). However, the entire continent of North America is predicted to remain climatically unfavorable except for the Gulf coastal plain and Atlantic coastal plains in the south and south‐eastern United States. Additionally, the modeling program predicts that the entire Florida peninsula, coastal plain of Texas and New Orleans to have a highly suitable climate to support the growth of *H*. *indica* (EI = 31–47). A large land area of South America, including Amazon basin is predicted to be suitable for *H*. *indica* (EI = 12–52) (Figure 2), whereas Piaui, central region of Brazil and part of eastern Columbia are predicted marginally suitable (18 < EI < 9). The western coastal plain of Peru, a large portion of southern cone that includes Chile and Argentina, western highland of Colombia and southern Venezuela are predicted to be unfavorable for species existence (EI = 0). In Africa, excluding southern cape, Somalia, northern Kenya, and Ethiopian highland, the entire sub‐Saharan along with eastern Madagascar are predicted to be moderately suitable or highly suitable to support the growth of *H*. *indica* (EI < 73) (Figure 3). Whereas most of the continental Europe, has climate conditions not conducive to *H*. *indica* (Figure 3). The only exception is the coastline of the Mediterranean basin which is predicted to have a marginally suitable climate to support *H*. *indica* growth (EI < 11).

**FIGURE 4:**
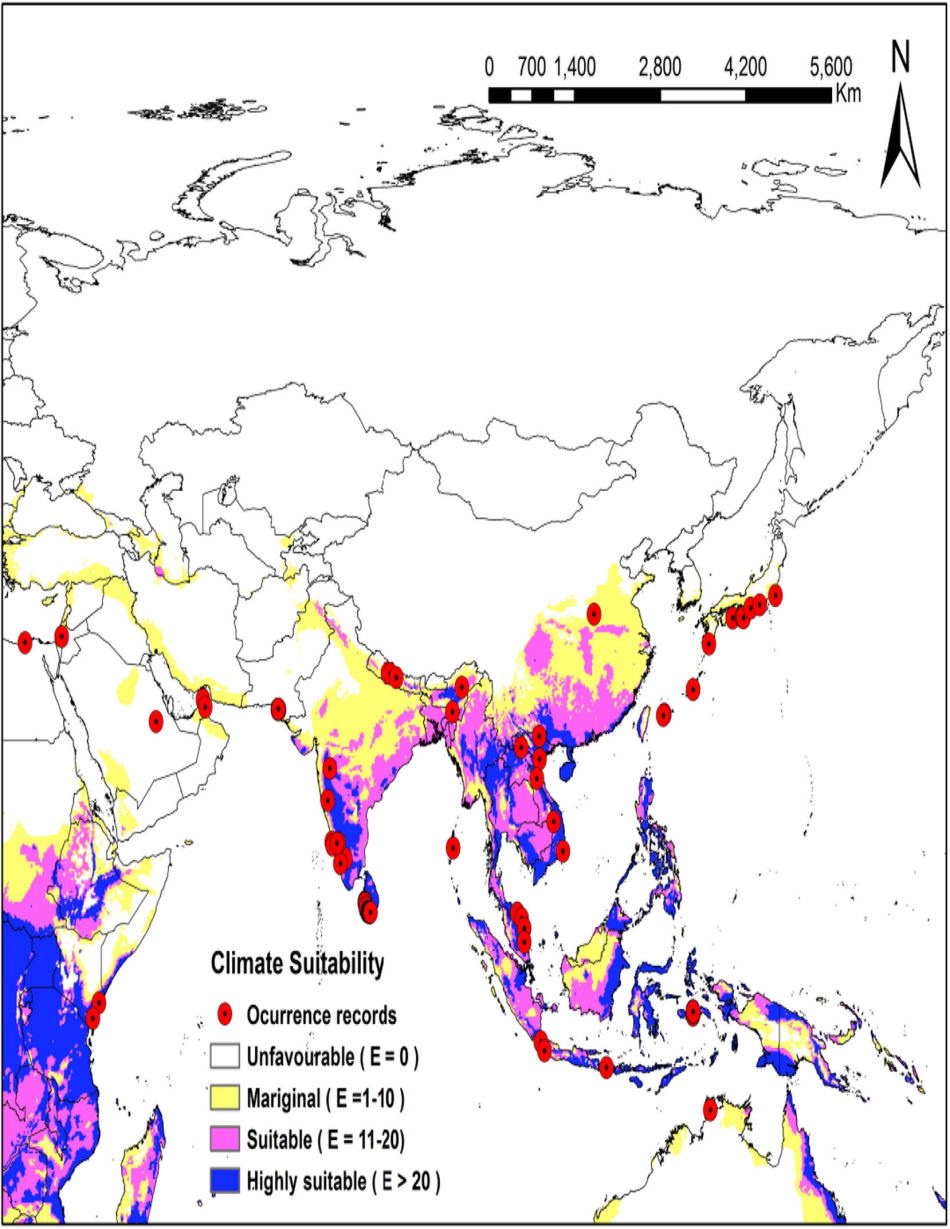
Ecoclimatic suitability (EI) for *Heterorhabditis indica* in Asia under the current climate scenario as predicted using CLIMEX. Red circles indicate occurrence records of *H*. *indica*

In Asia, the most suitable regions were confined to the Deccan plateau and Eastern Ghats of India (EI = from 28 to 58), Southeast Asia (EI < 72), south eastern China (EI < 32) along with a large portion of area in subtropical and warm temperate region of Japan (EI < 41) (Figure 4).

**FIGURE 5:**
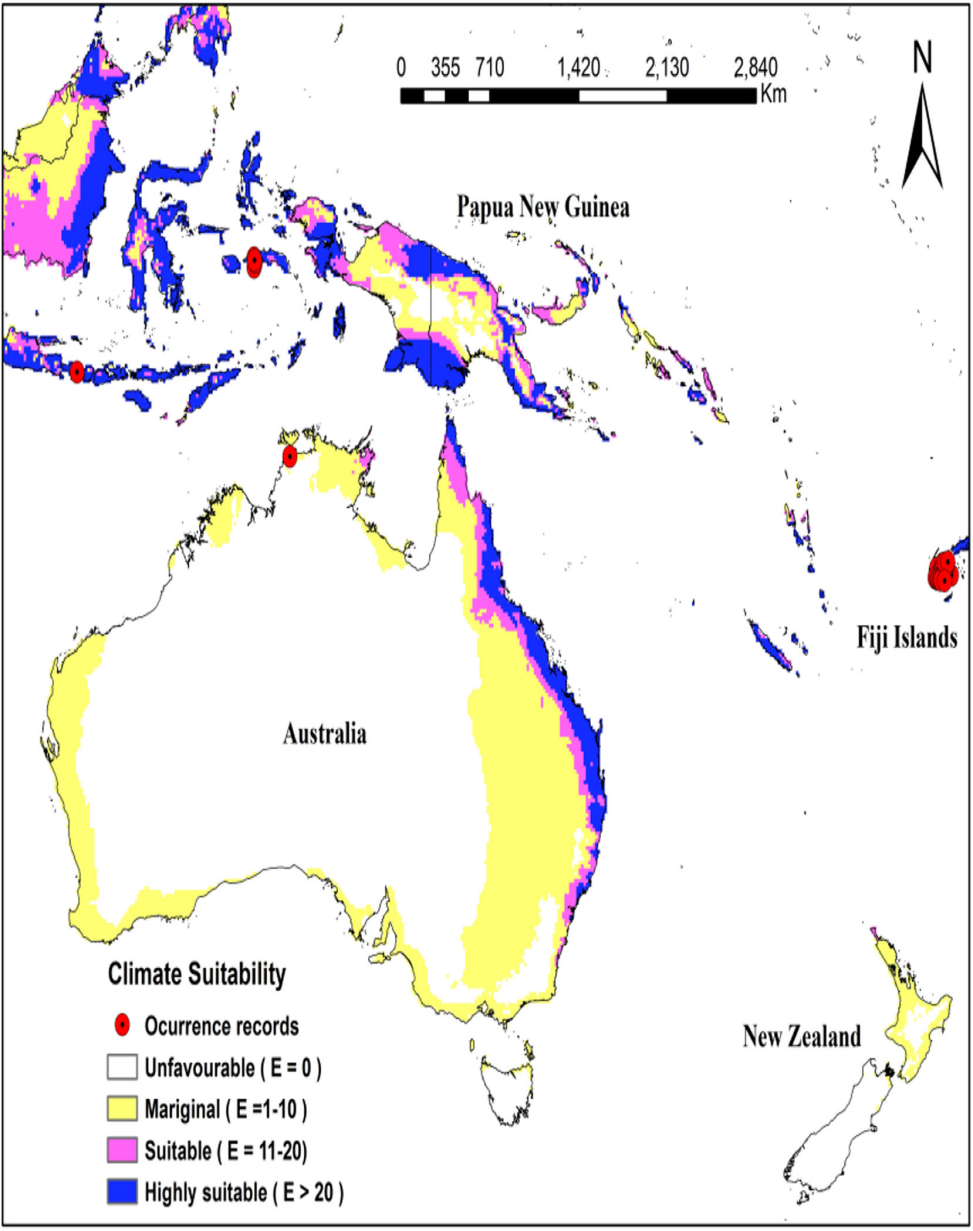
Ecoclimatic suitability (EI) for *Heterorhabditis indica* in Australia under the current climate scenario as predicted using CLIMEX. Red circles indicate recorded occurrence of *H*. *indica*

**FIGURE 6:**
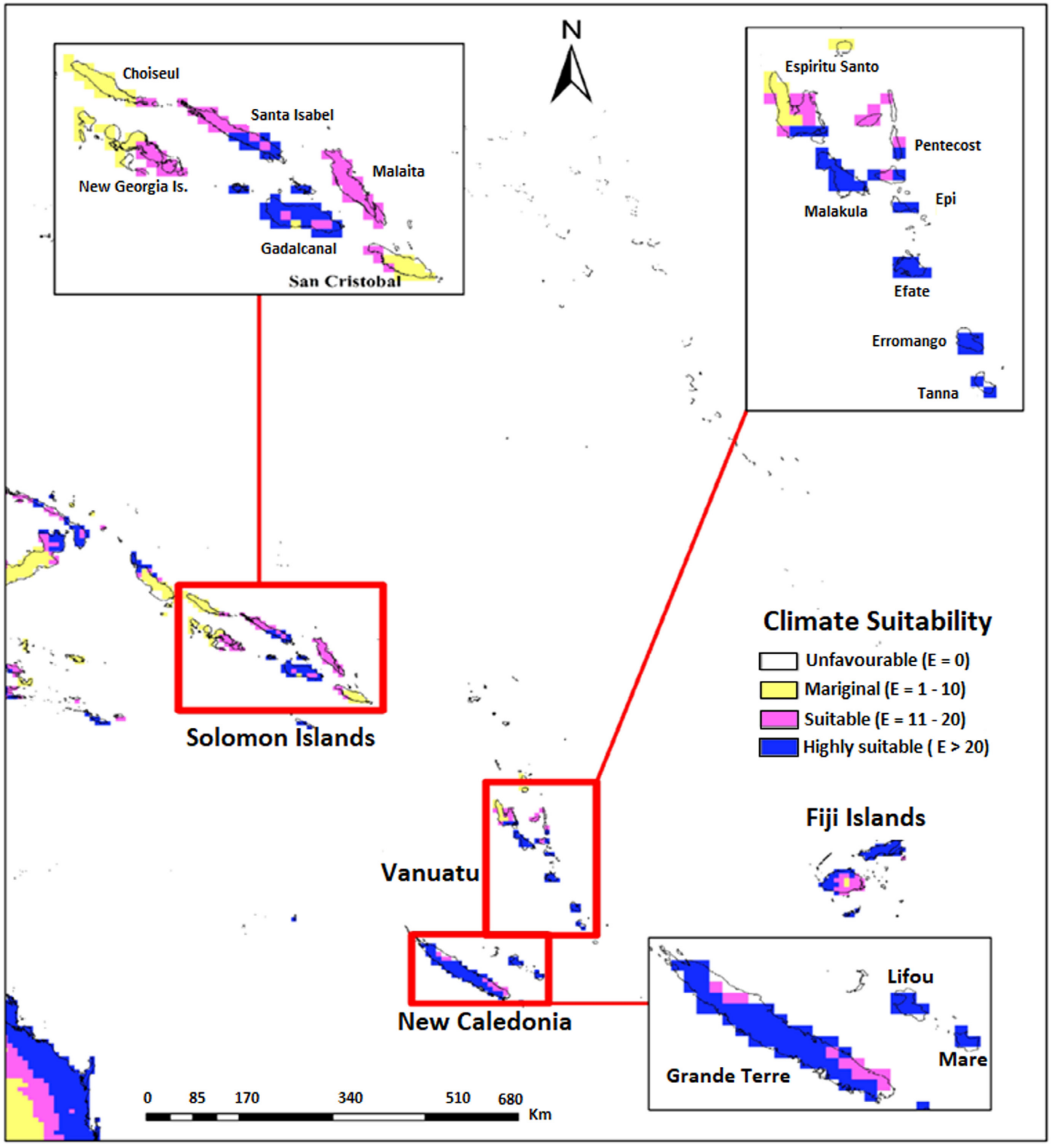
Ecoclimatic suitability (EI) for *Heterorhabditis indica* in Solomon Islands, Vanuatu and New Caledonia under current climate scenario as predicted using CLIMEX. Red circles indicate recorded occurrence of *H*. *indica*

**FIGURE 7:**
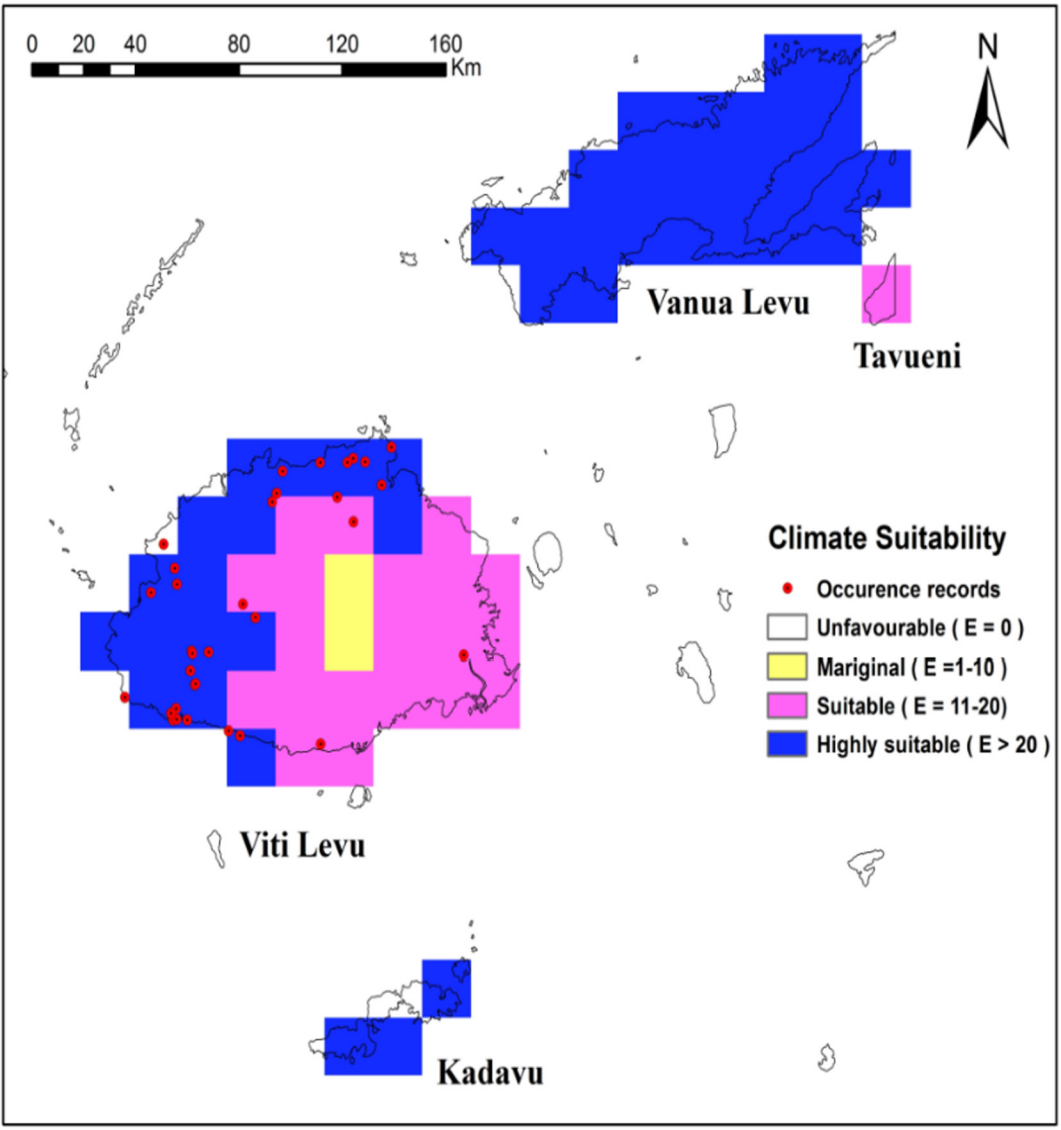
Ecoclimatic suitability (EI) for *Heterorhabditis indica* in Fiji Islands under the current climate scenario as predicted using CLIMEX. Red circles indicate recorded occurrence of *H*. *indica*

In the Pacific region, the north‐eastern tip of Australia including eastern coast that extends from Queensland to North South Wale (EI = 20–48) along with Hawaiian island (EI = 22–48) are climatically highly suitable (Figure 5). However, the rest of Australia and South Island of New Zealand are predicted to be climatically unsuitable for this species’ existence. Warm temperate regions of North Island of New Zealand have a very low EI values ranging from 0 to 5. The projected distribution of suitable habitat for *H*. *indica* in the South Pacific islands group is shown in Figures 6 and 7. All the major islands of New Caledonia (EI = 15–55), Vanuatu (EI = 10–44) except for western Espiritu Santo, and Solomon Islands (EI = 14–37) except for Choiseul, eastern San Cristobal and western New Georgia Islands, are predicted to be moderately suitable or highly suitable for *H*. *indica*. For Fiji Islands, the leeward side of the Viti Levu is predicted to be highly suitable for *H*. *indica* (EI = 20–51), whereas Windward side of Viti Levu is predicted suitable (EI = 14–20). Monasavu highland area is predicted to be only marginally suitable (EI = 8–10). Furthermore, entire Vanua Levu (EI = 23–41) and Kadavu (EI = 23–29) are shown to be climatically highly suitable and Taveuni (predicted EI = 20) to be climatically suitable for *H*. *indica*.

Figure 9 shows the predicted growth curves for *H*. *indica* in two localities in Fiji Islands, that is, Nadi and Suva. Upper portion of graphs shows average monthly temperatures and average monthly rainfall. Bottom portion of graphs depicts Temperature Index (TI) and Growth Index (GI) of *H*. *indica*. Graphs indicate that *H*. *indica* populations should decrease to low level during warmer and wetter months of the year indicating that in Viti Levu, summer months are stressful period for *H*. *indica*. In Suva, the species experiences longer stress period (Jan–April) than in Nadi (mid Jan–mid March). The maxima of growth curve for *H*. *indica* in Nadi is higher as compared to Suva. The CLIMEX output also predicts the dependence of growth index for *H*. *indica* on the rainfall. The rainfall has negative relation with the growth index.

**FIGURE 8:**
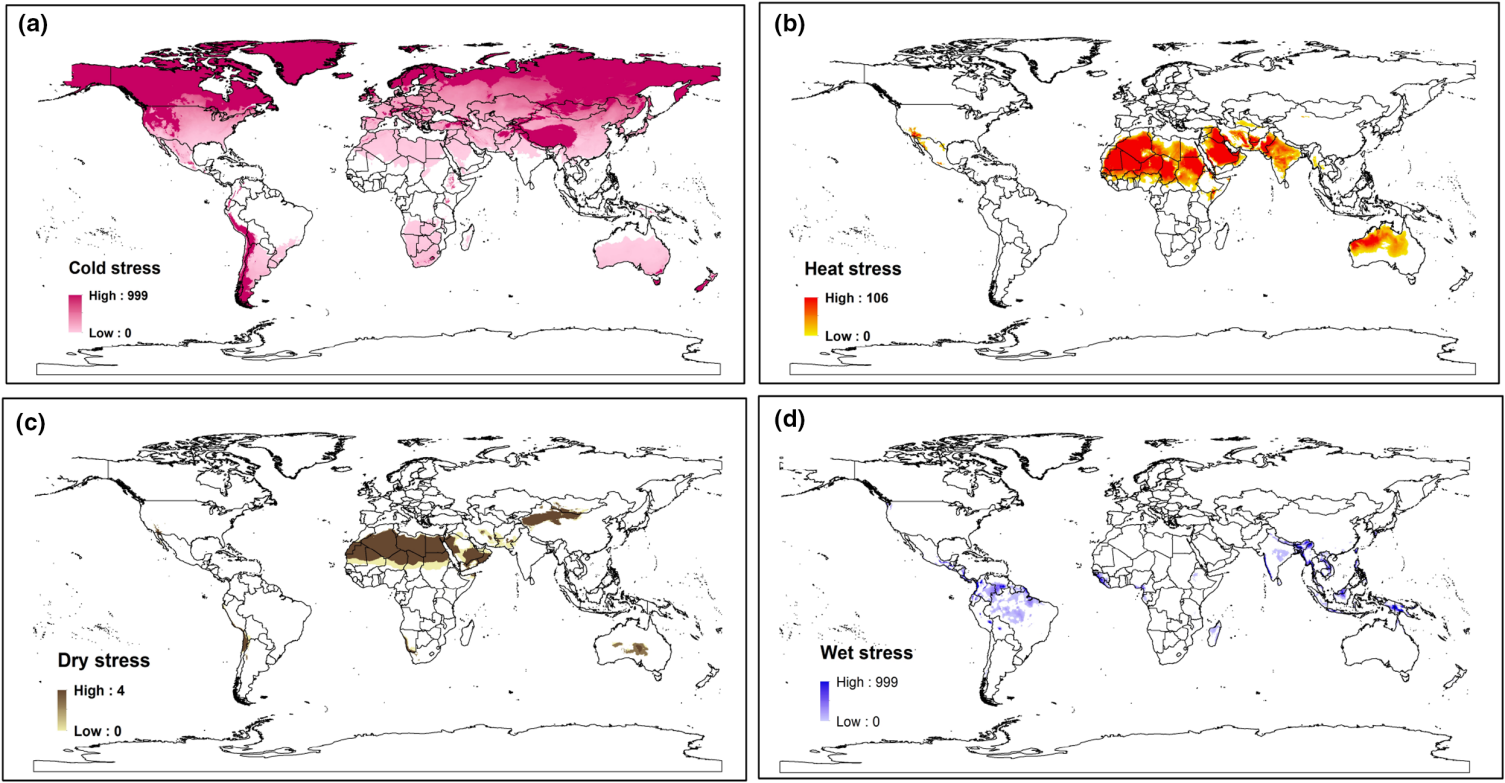
Predicted region where *Heterorhabditis indica* survival is affected by (a) cold stress, (b) heat stress, (c) dry stress, and (d) wet stress

Examination of the model output, predictions maps at a global level indicate boundaries of the potential climatic zone for *H*. *indica* is limited by cold stress, heat stress, and dry stresses in different area (Figure 8). However, cold stress appears to be the major limiting factor, whereas wet stress is the least limiting factor.

Projected land area that is climatically suitable for *H*. *indica* under the current climate was quantified for each region (Table 2). A total of 33% of the world's land mass (excluding Antarctica), or 3.60 million km^2^, is climatically suitable for *H*. *indica* survival. The results indicate that most parts (82%) of South America are climatically suitable, whereas the potential amounts of suitable areas in Europe and North America are the lowest at 15% and 11%, respectively.

**TABLE 2:** Projected land area (million km^2^) that is climatically suitable (EI ≥ 1) for *Heterorhabditis indica* under the current climate

Region	Area with EI ≥ 1 under historic climate	
	Total area (million km^2^)	% of total land area per region
Africa	16.8	57%
Asia	13.7	17%
North America	5.5	11%
South America	14.6	82%
Europe	0.9	15%
Oceania	2.8	33%
World (excluding Antarctica)	3.6	33%

## 4 DISCUSSION

Climate is one of the most important factors that strongly influence species distribution on a global scale. Earlier attempts to characterize climatic influences on species distributions, were explored by constructing climatographs (Messenger, 1976). Studies related to entomopathogenic nematodes distribution frequently employ statistical methods like correlation analysis to determine relationships between climatic variables and EPN occurrence. However, these methods have limitations because they only describe very simple relationships with one or two climate variables at a time. Multivariate statistical methods, such as logistic regression and principal component analysis (PCA) can often analyze large numbers of variables but rarely have been used to define distribution patterns of EPN apart from a few studies, for example, Kanga et al. (2012) used PCA to study the effect of climatic, geographical, and soil parameters on geographic spread of EPN in southern Cameroon. Campos‐Herrera et al. (2008) suggested the use of PCA to establish an environmental‐filtering model for EPN community. Stepwise multiple regression was also used by Campos‐Herrera et al. (2013) and Campos‐Herrera et al. (2016). Cluster analysis was used by Campos‐Herrera et al. (2007) to analyze effect of environmental variables on EPN distribution. Like simple correlation, multivariate techniques cannot explain complex interactions between a specie and the environment but do allow generalizations about which environmental factors may be more important in influencing the specie distribution. With the advent of computer technology and development of computer‐based simulation models, more complex analysis can be carried out for species and their relationships with climate (Senaratne et al., 2006). This study is the first to model the ecological niche of any EPN species and investigates the importance of environmental variables on projecting the range and spatial pattern of the modeled species *H*. *indica*. The only previous use of this approach for nematodes in general was by Porazinska et al. (2002).

An ecological niche model for *H*. *indica* predicts that, at the global level, the range for the distribution of suitable habitats of *H*. *indica* is mainly limited to subtropical, tropical, and coastline of warm temperate zones. The modeled climate suitability extends well beyond the current recorded occurrences. Generally, there is scarcity of data from actual surveys of EPN including *H*. *indica* and the projected distribution suggests that *H*. *indica's* actual distribution may be wider than the known distribution. A more systematic survey of areas that are predicted to be suitable for *H*. *indica* growth but still with no records can help in understanding the extent of distribution of this species. The global distribution of *H*. *indica* throughout the tropics, subtropics, and coastline of warm temperate zones highlights its large climate niche and broad climatic tolerance. *H*. *indica* possesses a range of phenotypic characters which give it an advantage in this climatic zone (Stack et al., 2000) and could be a potentially important nematode for the biological control of pest insects.

The model predicts that Africa and South America have the most suitable climate for the growth and establishment of *H*. *indica*. In both the continents, the species is expected to perform well under field conditions and has the potential to become a choice for use in biocontrol programs. However, both these continents remain largely unexplored for EPN occurrence. The same can be said for most of the south Pacific Islands to which are predicted to be moderately to highly suitable for *H*. *indica* but remain unexplored. The HSM developed in our current study can aid in prioritizing search areas for isolation of local populations of *H*. *indica* for use in biocontrol programs. Also, where countries import exotic species for biocontrol programs, it is best to select and obtain species or strains from areas with climatic conditions like that of the release area. Maladaptation of EPN species to the climate of the release area can be a major limiting factor to the success of a biological control programs (Haye et al., 2013; Hoddle et al., 2014). Since many of the commercially available EPN species are isolated from either North America or Europe (Grewal & Peters, 2005), it is important to know the release areas most suitable for their introduction. Therefore, it is important to develop models and generate HSM, for selecting the search areas where EPN might be found, or release areas most suitable for their introduction. The current model and the ecoclimatic and growth index output along with the habitat suitability map can be useful in identifying suitable areas where *H*. *indica* is likely to survive. The information can be useful in planning for identifying sites for sampling and also areas where *H*. *indica* can be used as a biological control agent on a global scale. Overlaying HSM for *H*. *indica* on the pest distribution map can further suggest whether the biocontrol agents be well suited to control the pest in new areas of application (Trethowan et al., 2011).

Under current climate scenario, the geographical range of *H*. *indica* appears primarily limited by cold stress and to a lesser extent by wet stress. The model suggests that northern range is strictly limited by the cold stress (Figure 8a) for this species. Cold stress appears to be the main limiting factor preventing *H*. *indica* from colonizing the northern temperate zone indicating that it is a warm‐adapted EPN and probably cannot tolerate long winter conditions in the cold regions. However, transient populations may possibly survive along coastline near Mediterranean basin where there are chances for marginal establishment as predicted by the model (Figure 3). Similarly, heat stress is suggested to be preventing its establishment in arid inland Australia, Middle East and Saharan Africa (Figure 8b). Saharan Africa as a habitat for *H*. *indica* also seems to be highly affected by dry stress (Figure 8c) and unlikely to support this species. If present at all, it would most probably be restricted to favorable microhabitats only such as the occurrence recorded from Saudi Arabia where *H*. *indica* has been reported from palm orchids. Otherwise, the arid land of Saudi Arabia comes up as unsuitable in CLIMEX modeling. In warm and arid regions, crops are predominantly under irrigated cultivation which may contribute to the successful potential establishment of *H*. *indica*. CLIMEX operates on a wider spatial and temporal climatic scale with less emphasis on microclimatic factors and hence is unable to detect microhabitats which are very important as well (Unwin & Corbet, 1991).

**FIGURE 9:**
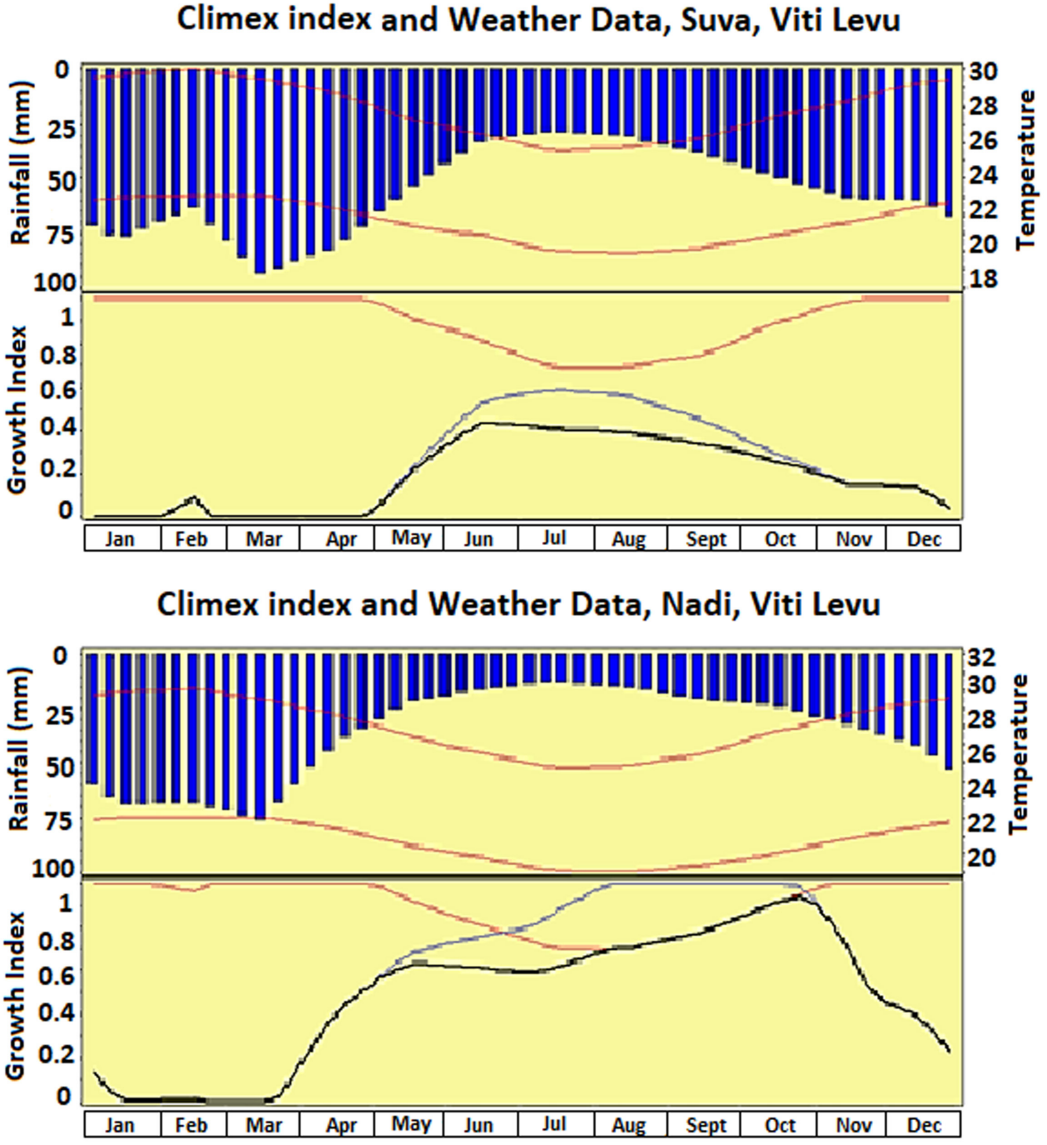
Modeled annual growth of *Heterorhabditis indica* in relation to the climate at two localities in Viti Levu

While the cold and dry stress conditions are the primary characteristics defining *H*. *indica* range frontiers, it is the rainfall and the soil moisture that characterize its population growth. Figure 9 shows the growth curve of *H*. *indica* at places in Viti Levu, Fiji Islands and clearly shows the effect of rainfall on population growth and EI values. This corresponds with the results of the survey reported by Kour et al., 2020. Results of this survey established that the occurrence of EPN in Viti Levu has significant negative association with the annual rainfall. The model predicts that a population in Suva remains at lower level for longer period (Jan–Apr) than in Nadi (mid Jan–mid Mar). This setback is due to the heavy rains in Suva from January till April. Suva (Windward side of Viti Levu) receives higher average rainfall than Nadi (leeward side of Viti Levu). Precise information, such as which season a species is likely to be active in, can be very useful for determining its use in pest management. The model findings confirm the most favorable time for the release of *H*. *indica*. The timing may be applicable to other EPN as well and can be confirmed from field studies. For most of its history, entomopathogenic nematology has been an applied discipline and has traditionally been more trial‐and‐error than predictive. This means, that no modeling of the potential impact of an agent is done before its release. Correct timing of introduction of biocontrol agents is therefore important for successful outcome. The model projections give an estimated likelihood of establishment of *H*. *indica*. However, there are other factors which influence the establishment and persistence of a species during broadcast application of EPN (Denno et al., 2008). EPN are applied inundatively to obtain immediate suppression of pest. Due to this the EPN application in inundative biological control is limited to high value crops. Large scale, outdoor agriculture crops are not treated although nematodes have the control potential (Susurluk & Ehlers, 2008). The cost benefit relation might change should EPN produce long‐term effects on pest populations. After application, establishment of biocontrol agent is the most desirable outcome and it will be more sustainable if EPN are able to establish themselves. To better understand how to establish sustainable nematode populations for pest management, the factors affecting of EPNs establishment must be understood (Alumai et al., 2006). This study further demonstrates the use of modeling in improving decision‐making in relation to EPN release strategy in biocontrol.

Models are generally calibrated with native range data or with combination of native and invaded range. This study used the global occurrence data to calibrate the model so that the full extent of the climatic conditions suitable for *H*. *indica* is captured. This results in increasing the sensitivity of the model (Webber et al., 2011). Model will underestimate the potential distribution of the target species if the parameters were fitted using the limited range. The validity of the model rests on how well the predictions match the reality. The map comparisons show good agreement between simulated and present distributions of *H*. *indica*, indicating that the climate base models can provide useful predictions with minimum input information. However, being climate based, the model completely ignores nonclimatic factors that also determine habitat suitability for a particular species (Kriticos et al., 2003). There are many biotic and abiotic factors that affect EPN geographical distribution (Hominick et al., 1996). Other factors such as soil properties and biotic interactions may prevent species from colonizing sites that are otherwise climatically suitable. Inclusion of soil type and host distribution into the climate projections will further improve the predictive ability of CLIMEX output.

CLIMEX has been used in various aspects of species distribution modeling and has been shown to have predictive accuracy across broad geographic and taxonomic scales. In this study, the software was applied for first time as a tool to model ecological niche of *H*. *indica*. Since the distribution is modeled at the species level, the habitat suitability projections can be used to identify where the species could establish as a biological control agent. The CLIMEX model output provides values for locations on a fine scale globally for growth and eco‐climatic suitability which is used to generate the habitat suitability maps. The high accuracy of CLIMEX predictions of known distribution and effect of environmental factors on *H*. *indica* growth suggest that this modeling approach holds great promise in developing the biocontrol programs.

## 5 LIMITATION

In pest management, the benefits and dangers of using modeling will not be fully appreciated until limitations of specific models are discussed openly (Worner, 1991). Modeling the potential distribution of a species using climatic mapping has received some criticism because the potential distributions cannot be predicted based on climate alone (Japoshvili et al., 2015; Soberon & Peterson, 2005). Biotic interactions, such as host availability, soil type, geographical barriers to dispersal, are also limiting factors in species distribution (Legaspi & Legaspi, 2010; Ni et al., 2012; Zalucki & Van Klinken, 2006). However, we consider that the method described herein is a very useful predictive tool and should be seen as “First approximations” of species distribution (Pearson & Dawson, 2003).

## 6 CONCLUSION

Despite the popularity of Habitat Suitability Maps (HSM), this method has never been used to model EPN species. In this study, we highlighted an efficient way to construct HSM for *H*. *indica*. The derived model was used to develop distributional maps, potentially useful in the search/release of target species in new locations. The study showed that *H*. *indica* can potentially establish itself throughout much of the tropics and subtropics and also the warm temperate region. For best results, the release timing of EPN should be adjusted with season for maximum growth. This modeling data enhances the value of actual EPN distribution mapped in this study and presents an opportunity for designing potentially successful biocontrol trials.

It is first time that an Ecological Niche Model for *H*. *indica* was generated using CLIMEX software, which led to generation of Habitat Suitability Map (HSM) predicting it is the global habitat distribution. *H*. *indica* is first EPN to be modeled thus. This predictive model can be very useful both for exploring these habitats for *H*. *indica* and/or in selecting the release area for application in biocontrol.

## CONFLICT OF INTEREST

The authors declare that they have no known competing financial interests or personal relationships that could have appeared to influence the work reported in this paper.

## AUTHOR CONTRIBUTIONS

**Sumeet Kour:** Conceptualization (equal). **Uma Khurma:** Conceptualization (equal). **Gilianne Brodie:** Conceptualization (equal). **Sunil Singh:** Conceptualization (equal).

## Data Availability

Modeling the potential global distribution of suitable habitat for the biological control agent Heterorhabditis indica Dyrad: doi: https://datadryad.org/stash/share/uGH72BSAowtF3_ELPcxboV1Jpi1JbhtZivrWRV9fKAA
